# Draft Genome Sequence of *Exiguobacterium* sp. Strain N5, Isolated from a Recreational Freshwater Kettle Lake in Ontario

**DOI:** 10.1128/mra.01261-22

**Published:** 2023-03-07

**Authors:** Noah Bryan, Rebecca Anderson, Opeyemi U. Lawal, Valeria R. Parreira, Lawrence Goodridge

**Affiliations:** a Canadian Research Institute for Food Safety (CRIFS), Department of Food Science, University of Guelph, Guelph, Ontario, Canada; b Bayview Secondary School, Richmond Hill, Ontario, Canada; University of Delaware College of Engineering

## Abstract

*Exiguobacterium* spp. are facultative anaerobic, Gram-positive, non-spore-forming bacilli, reported to tolerate extreme environments. Here, we report the draft genome sequence of *Exiguobacterium* sp. strain N5, isolated from a recreational freshwater lake.

## ANNOUNCEMENT

*Exiguobacterium* spp. are facultative anaerobic, Gram-positive bacilli known for their capability to survive in extreme environments (1–3). *Exiguobacterium* sp. strain N5 was isolated from a recreational freshwater kettle lake in Richmond Hill, Ontario, Canada (43.9486°N, 79.4352°W). Freshwater samples (1 L) were collected from shallow regions of Lake Wilcox, adjacent to the shore, and kept at 4°C until processing. Samples (1 mL) were serially diluted in 9 mL of modified saline-magnesium (SM) buffer (without gelatin) (4) and plated onto tryptic soy agar (TSA). The plates were incubated for 24 h at 37°C. Bacterial colonies of differing morphologies were subcultured on TSA. Genomic DNA from a pure colony was extracted using the DNeasy blood and tissue kit (Qiagen, Hilden, Germany) according to the manufacturer’s instructions. DNA libraries were prepared using the Illumina DNA prep tagmentation kit (catalog number 20018704) and IDT for Illumina DNA/RNA unique dual (UD) indexes (catalog number 20027213). Paired-end (2 × 150-bp) sequencing was performed on the Illumina MiniSeq system. The raw paired-end reads were quality filtered using FastQC v0.11.9 (https://github.com/s-andrews/FastQC) and trimmed using Trimmomatic v0.39 (5). Reads with Phred scores above 20 were assembled *de novo* using SKESA v2.4.0 (6). The assembly quality was assessed using QUAST v5.2 (7). Genome annotation was performed using the NCBI Prokaryotic Genome Annotation Pipeline v6.3 (8). Resistomes were determined using CARD (9) and AMRFinder Plus v3.10.45 (10), while the virulome was identified using VFDB (11, 12). The draft genome was screened for plasmids and prophages using MOB-suite v3.1 (13) and PHASTER (14), respectively. Biosynthetic gene clusters were assessed using antiSMASH v6 (15). All bioinformatics tools were used with default parameters.

**FIG 1:**
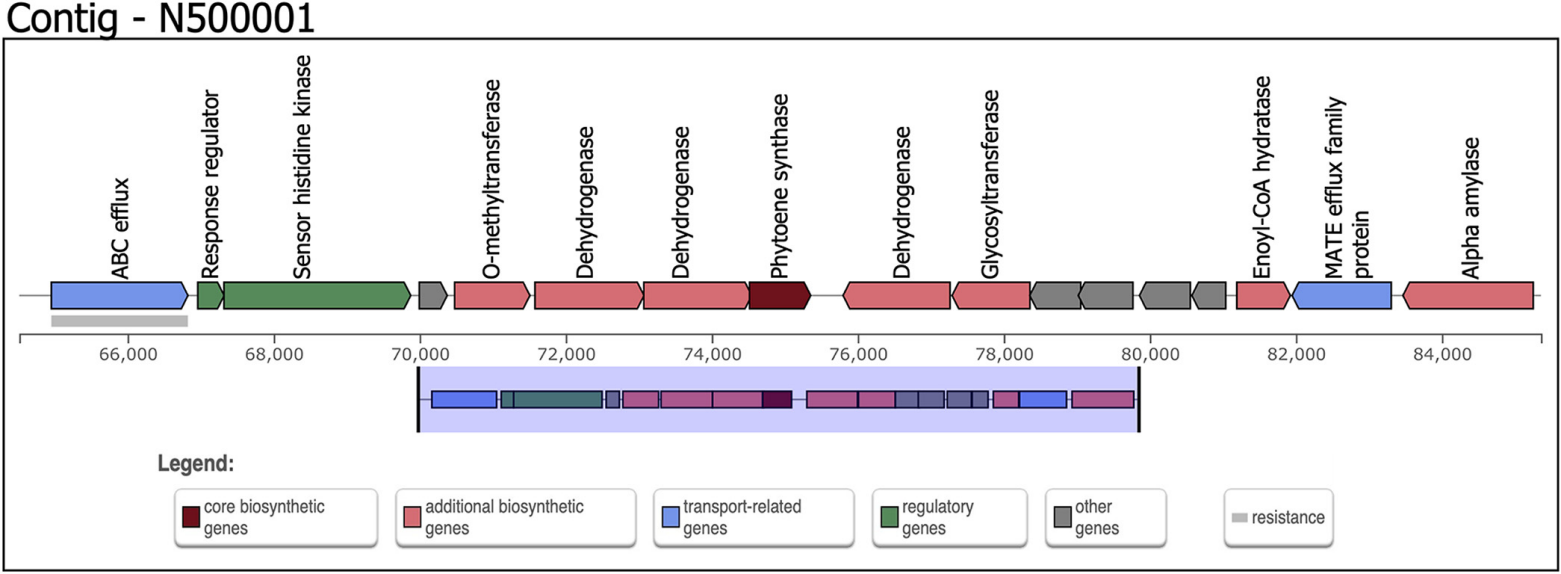
Structure of the terpene biosynthetic gene cluster found in *Exiguobacterium* sp. strain N5. The draft genome was screened for the presence of biosynthetic gene clusters using antiSMASH v6 (https://antismash.secondarymetabolites.org).

Sequencing of isolate N5 yielded 2,484,695 paired-end raw reads. Isolate N5 was identified as *Exiguobacterium* sp. using *k*-mer-based species identification with the Kraken 2 database (16, 17). Genome assembly revealed a genome size of 2,912,151 bp and 14 contigs with an *N*_50_ value of 2,062,816 bp, 219× mean coverage, and 47.85% GC content. This strain contained 2,994 protein coding sequences and 63 tRNAs. A comparative analysis between this strain and previously sequenced *Exiguobacterium* isolates revealed 95.54% average nucleotide identity (ANI) with *Exiguobacterium* sp. strain AT1b (GenBank accession number CP001615.1) using ANItools (18, 19). Plasmids and genes encoding virulence and antimicrobial resistance were not detected. The only phage region identified (contig N500001; position 1304909 to 1332480) was an incomplete phage without an integrase gene. Additionally, the draft genome contained a terpene biosynthetic gene cluster (contig N500001; position 64517 to 85356) that had 78% homology with the terpene cluster found in Halobacillus halophilus DSM 2266 (NC_017668.1). The gene cluster was flanked by transport-related genes (ABC and MATE efflux family proteins). Within the gene cluster was a phytoene synthase gene, with additional biosynthetic genes upstream and downstream of it encoding O-methyltransferase, dehydrogenases, and glycosyltransferase. Regulatory genes encoding a sensor histidine kinase and response regulator were also found upstream of the phytoene synthase gene (Fig. 1).

### Data availability.

This whole-genome shotgun project has been deposited at DDBJ/ENA/GenBank under the accession number JAPAEP000000000, and the raw reads were deposited under SRA accession number SRR22013159. The version described in this paper is version JAPAEP000000000.
